# Comparative Analysis of Red Onion-Derived Exosome-Like Nanovesicles and Extract Reveals Sustained Immunomodulatory Effects in LPS/IFN-γ-Stimulated Microglia

**DOI:** 10.1007/s12035-026-05820-0

**Published:** 2026-03-30

**Authors:** Sedra Alhelwani, Nurdan Sena Degirmenci, Cihan Mehmet Altıntaş, Mehtap Aydın, Fikrettin Şahin, Mehmet Şenel, Sevim Işık

**Affiliations:** 1 Department of Molecular Biology, Institute of Science, Uskudar University, Istanbul, 34662 Turkey; 2 Department of Molecular Biology and Genetics, Faculty of Engineering and Natural Sciences, Uskudar University, Istanbul, 34662 Turkey; 3 Stem Cell Research and Application Center (USKOKMER), Uskudar University, Istanbul, 34662 Turkey; 4 Department of Genetics and Bioengineering, Faculty of Engineering, Yeditepe University, Istanbul, 34755 Turkey; 5 SANKARA Brain and Biotechnology Research Center, Istanbul, 34320 Turkey; 6 Department of Molecular Biology and Genetics, Faculty of Engineering and Natural Sciences, Istanbul Sabahattin Zaim University, Istanbul, 34303 Turkey; 7 Department of Biochemistry, Faculty of Pharmacy, Biruni University, 34010 Istanbul, Turkey

**Keywords:** Red onion–derived exosome-like nanovesicles, Phyto-nanotechnology, Extract, Neuroinflammation, NLRP3 Inflammasome, Microglia

## Abstract

Neuroinflammation is a central factor in many neurodegenerative diseases, making it an important target for novel treatments. This study compares the effects of organic red onion–derived materials, particularly crude extract and exosome-like nanovesicles (RO-ELNs), on neuroinflammation in the human microglia HMC3 cell line. Red onions are abundant in neuroprotective phytochemicals, primarily quercetin and anthocyanin constituents. The non-cytotoxic RO-ELNs purified through ultracentrifugation, along with their corresponding ethanolic extract, showed that they contain inherited phytopharmaceutical compounds from their parent source. Despite RO-ELNs having lower metabolite concentrations relative to the extract, their therapeutic value is mostly attributed to their lipids and other components. Both treatments engage similar biological pathways yet demonstrate contrasting time-dependent anti-neuroinflammatory and antioxidant effects by suppressing NLRP3 inflammasome components (NLRP3, IL-1β) and critical inflammatory markers (COX-2, phospho-NF-κB, ROS, IL-6, TNF-α) during acute inflammation. Nevertheless, the extract showed restricted effectiveness in prolonged inflammation or at longer incubation times owing to its poor bioavailability. Conversely, RO-ELNs showed a robust and lasting effect due to their lipid bilayer, facilitating gradual uptake and extended retention within target microglia, with uptake escalating from 20 to 70% over 24 to 72 h compared to control. This study uniquely investigates the time-dependent anti-inflammatory and antioxidant actions of red onion–derived ELNs and extract across acute and prolonged phases of neuroinflammation, suggesting RO-ELNs as a promising nanosized approach for neuroinflammation prevention.

**Graphical Abstract:**

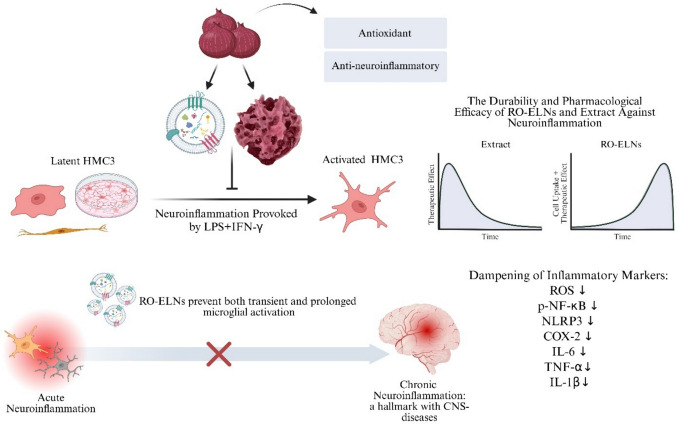

**Supplementary Information:**

The online version contains supplementary material available at 10.1007/s12035-026-05820-0.

## Introduction

Neuroinflammation, a complex response involving activation and recruitment of central nervous system (CNS) immune cells, is triggered by factors like infections, injuries, toxins, or trauma. It releases pro-inflammatory mediators as a defense mechanism like cytokines, chemokines, and reactive oxygen species (ROS), which contribute to oxidative stress and neuronal damage [8]. Importantly, neuroinflammation contributes to neurodegenerative diseases (NDDs) progression such as Alzheimer’s disease (AD) and Parkinson’s disease (PD) by promoting neuronal injury and synaptic dysfunction [23]. Neuroinflammation and oxidative stress mutually reinforce each other, causing mitochondrial damage, redox homeostasis imbalance, the buildup of neurotoxic metabolites, and indirectly promoting misfolded protein aggregates like amyloid-beta (Aβ) and alpha-synuclein (α-syn), which increase blood-brain barrier (BBB) permeability and infiltration [53, 54]. Microglia, CNS-resident macrophages, shift from resting to activated state when encountering toxins, migrating and phagocytizing damaged cells [52]). Bacterial lipopolysaccharides (LPS) bind to toll-like receptor 4 (TLR4) on microglia, triggering innate immune responses via downstream signaling pathways like NF-κB [5], which promotes the upregulation of pro-inflammatory genes (e.g., NLRP3, COX-2) and release of interleukin-1 beta (IL-1β), interleukin-6 (IL-6), tumor necrosis factor-alpha (TNF-α), and ROS, leading to neurotoxicity [6]. Microglial overactivation without effective clearance drives a shift from acute to chronic inflammation, marked by persistent oxidative stress, cytokine release, and recruitment of adaptive immune responses, ultimately impairing BBB integrity and neurophysiology [48, 49]). Collectively, this neuroinflammatory network leads to neurodegeneration [15]. Although targeting these pathways may reduce inflammation and protect the brain, existing anti-inflammatory treatments like NSAIDs and IL-1 inhibitors are limited by poor BBB penetration, high costs, frequent dosing, and side effects [39, 47]. Overall, synthetic drugs mainly slow disease progression and relieve symptoms but do not provide a definitive cure, posing an ongoing public health challenge.

Harnessing herbal sources such as *Allium cepa *L. for their antioxidant and anti-inflammatory compounds is a critical therapeutic approach investigated in neuronal disease research [12, 19,14 ,). Onions are important agricultural crops and are renowned for their distinct flavor, natural sugars, and numerous health benefits [18]. They are rich in essential nutrients, including potassium, zinc, vitamins, fiber, and polyphenols, including flavonoids like quercetins and their derivatives, as well as kaempferol, isorhamnetin, and myricetin [34]. These flavonoids endow onions with potent antioxidant, anti-inflammatory, antibacterial, antifungal, anti-platelet, and anti-hyperlipidemic properties, contributing to the pharmacological advantages of onions [35, 45]. Red or purple onions are distinguished by their high anthocyanin content, correlating with their strong antioxidant properties [28]. Specifically, the unique chemical structures of quercetin and anthocyanins promote health [56].

Developing herbal medicine sources into effective biomaterials that could serve as drug delivery vehicles and cross biological barriers is needed for novel and precise therapeutic approaches [41]. Plant-derived exosome-like nanovesicles (ELNs), which are biological nanostructures surrounded by a lipid bilayer, measuring approximately 50–200 nm in diameter, are secreted into the extracellular space to transport proteins, lipids, DNAs, and RNAs to other cells [44]. Plant ELNs facilitate beneficial inter-kingdom communication and human health by delivering encapsulated source-inherited enzymatic antioxidants and phytochemicals protected by their lipid bilayer architecture [27, 38]. Plant ELNs offer a scalable, biocompatible, and cost-effective alternative to mammalian exosomes and artificial nanocarriers, with minimal immunogenicity. Their strong lipid membranes confer high biostability, enabling resistance to environmental stresses and effective delivery through gastric and intestinal fluids, including crossing the BBB for treating neuroinflammatory and NDD [22]. Other previous research on plant ELNs, such as ELNs from *Allium tuberosum*, *Atractylodes lancea*, and oat, has shown potential in suppressing microglial activation and reducing neuroinflammation [21, 26, 51].

Therefore, we hypothesize that ELNs, due to their lipid bilayer, provide enhanced protection and transport of anti-inflammatory and antioxidant agents to microglia. The lipid bilayer is expected to improve cellular absorption, resulting in a more efficient attenuation of LPS/IFN-γ-induced inflammation than the extract. This work investigates the defensive role of exosome-like nanovesicles (RO-ELNs) and crude extracts from edible red onions in regulating neuroinflammation. Microglial cells were pretreated with RO-ELNs and extract prior to LPS/IFN-γ exposure to evaluate their impact on both acute (early) and prolonged inflammation. To our knowledge, this is the first direct comparison of plant-derived ELNs and extracts concerning neuroinflammation.

## Materials and Methods

### Processing of Red Onion ELNs and Extracts

Organic Red Onions (*Allium cepa *L.) were purchased from the local farmers’ market, washed with distilled water, and ground using a blender to obtain homogenates.

To isolate and purify exosomes (ELNs), homogenates were mixed with distilled water and squeezed to recover the juice. The juice was then sequentially centrifuged at 3600 × g for 10 min at 4 ℃. Then, 10,000 × g for 20 min at 4 ℃ where the pellet was discarded each time. Then, the recovered supernatant was passed through a 0.45-µm syringe filter. The samples were then transferred into thin-wall ultra-clear centrifuge tubes with 1 molar of sucrose added to the bottom of the tubes to create a visible interphase/density gradient and ultracentrifuged at 100,000 × g for 1 h at 17 ℃ to fractionate the ELNs. Next, the obtained ELNs within sucrose were washed with distilled water to remove sucrose using a 100-kDa MWCO ultrafiltration centrifuge tube at 3600 × g for 12 min at 4 ℃. The final concentrated collected ELNs were resuspended in PBS and passed through a 0.22-µm syringe filter.

To prepare the extracts, ethanolic extraction was performed by mixing 200 g (150 mL) of red onion homogenates/puree with 300 mL absolute ethanol at a 1:2 (w/v) ratio in a beaker and stirring the mixture at room temperature using a magnetic stirrer for 4 h. Then, the mixture was filtered through Whatman No. 42 filter paper, and the volume was reduced to approximately 230–250 mL. The filtrate was concentrated at 45 ℃ with a rotary evaporator, thereafter lyophilized to provide purplish-red syrupy residues totaling approximately 25 mL, mainly including non-volatile constituents with the ethanol levels being negligible. The treatment stock concentration was prepared by dissolving the residues with PBS in 300 mg/ml and passed through a 0.22-µm syringe filter.

### Characterization of ELNs

Nanoparticle tracking analysis (NTA) was conducted on isolated exosomes (ELNs) through a NanoSight NS300 device (Malvern Panalytical). The ELNs were diluted at a ratio of 1:1000 in double-distilled water. The distribution of the particle sizes and concentration of the exosome samples were assessed using the NTA 3.3 Software Suite. All measurements were conducted at room temperature, with each sample examined in triplicates. Additionally, for zeta potential (ζ-potential) analysis, the ELNs were similarly diluted in double-distilled water at 1:1000. Dynamic light scattering (DLS) measurements were then performed at 25 °C using a Nano Zeta sizer [24]. Furthermore, ELNs isolated from red onions were also analyzed using atomic force microscopy (AFM), following the established imaging protocols outlined in prior studies.

[4].

LC-20A HPLC system (Kyoto, Japan) was employed to analyze anthocyanins and quercetin in red onion–derived samples, including juice, extracts, and exosomes (ELNs), using two different column configurations. Samples of juice and extract were filtered through a 0.45-μm syringe filter and dissolved in a 70% methanol solution (1:2 ratio) prior to analysis. To extract flavonoids from ELNs, 200 μL of concentrated ELNs obtained directly after ultracentrifugation were subjected to sonication in a water bath at 50 ℃ for 25 min and then mixed with 200 μl of 70% methanol. For compounds not represented by additional standards, we referenced established literature to accurately identify and confirm the retention times and patterns of the remaining peaks [34].

For anthocyanins and phenolic compounds analysis, a 5-μm Inertsil ODS-3 reversed-phase column (250 × 4.6 mm; GL Sciences) was used. Samples were injected at 20 μL into the HPLC-DAD system. Separation was performed with a mobile phase consisting of solvent A (water with 7% formic acid) and solvent B (acetonitrile, water, and methanol in a 90:5:5 ratio) under a gradient condition at a flow rate of 1.2 mL/min, with the column maintained at 25 °C. Detection was carried out at 520 nm to identify anthocyanins.

### Fluorescent Labeling and Intracellular Uptake of ELNs

According to the manufacturer’s protocol, RO-ELNs were labeled with CM-DiD dye (22033, AAT Bioquest, Pleasanton, CA, USA). First, 5 µM was added to 400 µl of isolated RO-ELNs with 50 µl PBS and incubated for 30 min at 37 ℃. Then a 100-kDa MWCO ultrafiltration centrifuge tube was used to collect the labeled RO-ELNs at 3000 g for 30 min. The labeled RO-ELNs were incubated with HMC3 at several points: 3, 6, 24, 48, and 72 h. HMC3 cells were washed with PBS, stained with DAPI, and imaged using a fluorescent microscope.

### HMC3 Cell Culture

Human microglial cell line (HMC3) was purchased from the American Type Culture Collection (ATCC, USA. CRL-3304). The HMC3 cells were cultured in minimum essential medium (MEM; Capricorn, MEM-XA), supplemented with 10% fetal bovine serum (FBS; Gibco, 10,270,106), 1% pen/strep (penicillin/streptomycin; Capricorn, PS-B), 1% L-glutamine (Capricorn, GLN.B), and 1% sodium pyruvate (Capricorn, NPY.B), and cells were grown at 37 ℃ with 5% CO_2_.

### Cytotoxicity and Cell Proliferation Assay

Cell viability was assessed using the MTT assay. HMC3 cells were seeded in a 96-well plate at a density of 7.5 × 10^3^ cells per well and cultured at 37 ℃ with 5% CO_2_ overnight. The following day, first, the cells were treated with RO-ELNS or extracts for 24 h to evaluate their optimal concentration. Second, cells were pretreated with RO-ELNs for either 6 h or 20 h and extract for 6 h followed by 50 µM of ATP (Thermo Fisher, R0441) for 10 min then 1 µg/mL of LPS (Sigma-Aldrich, L4391-1MG) and 0.01 µg/mL of IFN-γ (Gibco, PHC4031) for either 4 h or 48 h. After the treatment period, the old medium was replaced with fresh medium, and 10 µL of MTT solution (3-(4,5-dimethylthiazol-2-yl)−2,5-diphenyltetrazolium bromide; Roche, Cat# 11,465,007,001) was added to each well. The cells were incubated for 4 h at 37 °C. Subsequently, 100 µL of solubilization buffer was added to each well and incubated for 16 h. The remaining step involved measuring the absorbance at 570 nm using a microplate reader to evaluate cell viability.

### Intracellular ROS Production Assay

Reactive oxygen species levels were assessed using the DCFH-DA assay. HMC3 cells were seeded in 96-well plate at a density of 7.5 × 10^3^ cells per well and cultured at 37 ℃ with 5% CO_2_ overnight. The following day, the cells were pretreated with RO-ELNs for either 6 h or 20 h and extracts for 6 h followed by stimulation with 50 µM of ATP for 10 min to activate the inflammasome. The cells were then primed with 1 µg/mL of LPS 0,01 µg/mL of IFN-γ for either 4 h or 48 h depending on the severity of neuroinflammation model being investigated. Following the treatment, the cells were rinsed thoroughly with PBS. Subsequently, a fresh culture medium containing 20 µM DCFH-DA dye was applied to each well, and the plates were incubated at 37 °C for 30 min. After this incubation period, the cells were washed once more with PBS to eliminate any remaining dye. The fluorescence signal was then detected by measuring excitation/emission wavelengths of 485/535 nm with a microplate reader.

### Immunoblot Analysis

HMC3 cells were supplemented with RO-ELNs and extracts and incubated with LPS/IFN-$\gamma$+ATP. Cells were lysed with RIPA buffer, and the protein concentration was measured using BCA assay. Fifty micrograms of proteins was separated by SDS-PAGE. The proteins were then transferred to a PVDF membrane at constant current 1 A, and the voltage was variable limited to 25 V for 70 min. After blocking with 5% skim milk, the membranes were then incubated with NLRP3 (Invitrogen, MA5-32255), COX-2 (Invitrogen, 35–8200), phosphorylated NF-κB (p-p65) (Cell Signaling. 3033), and GAPDH (Boster, M00227-7) antibodies overnight at 4 ℃. After washing, membranes were incubated with an anti-mouse IgG with horseradish peroxidase (HRP) (Abcam, ab97040), or anti-rabbit IgG with horseradish peroxidase (HRP) (Abcam, ab97080). Western Bright ECL-HRP substrate (Advansta, ADV-K-12045-C20) was used for the chemiluminescence reaction, and the membranes were visualized on the C-Digit Blot Scanner (LICOR) device.

### Inflammatory Cytokine Detection by LEGENDplex™ Assay

In this research, levels of the inflammatory cytokines IL-6, IL-1β, and TNF-α were measured utilizing the LEGENDplex™ Human Essential Immune Response Panel (BioLegend, USA), following the instructions provided by the manufacturer. To perform the assay, 25 µL of cell supernatant samples, 25 µL of mixed capture beads, and 25 µL of assay buffer were combined in each well of a filter plate and then incubated for 2 h at room temperature on a shaker set to 500 rpm in the dark. After incubation, the plate was washed twice with 1× wash buffer using a vacuum filtration system. Subsequently, 25 µL of biotinylated detection antibody was added to each well and incubated for 1 h under the same conditions. Without additional washing, 25 µL of streptavidin-phycoerythrin (SA-PE) was added, followed by another 30-min incubation. After the final wash step, 150 µL of 1× wash buffer was added to resuspend the beads. The samples were then analyzed via flow cytometry, and IL-6, IL-1β, and TNF-α concentrations were determined using the QuantiGlo software.

### Statistical Analysis

The results are presented as the mean ± standard deviation (SD) for data obtained from two or three independent experiments. The data was assumed to follow a normal distribution based on experimental design and prior research. Statistical analysis was applied as appropriate using one-way and two-way analysis of variance (ANOVA). Post hoc multiple comparisons were conducted using Tukey’s or Sidak’s tests with a significance level set at *p* < 0.05. All assays were conducted in duplicate-triplicate before being reported. The data were analyzed using GraphPad Prism (version 8.0).

## Results

### Isolation and Characterization of RO-ELNs

**Fig. 1:**
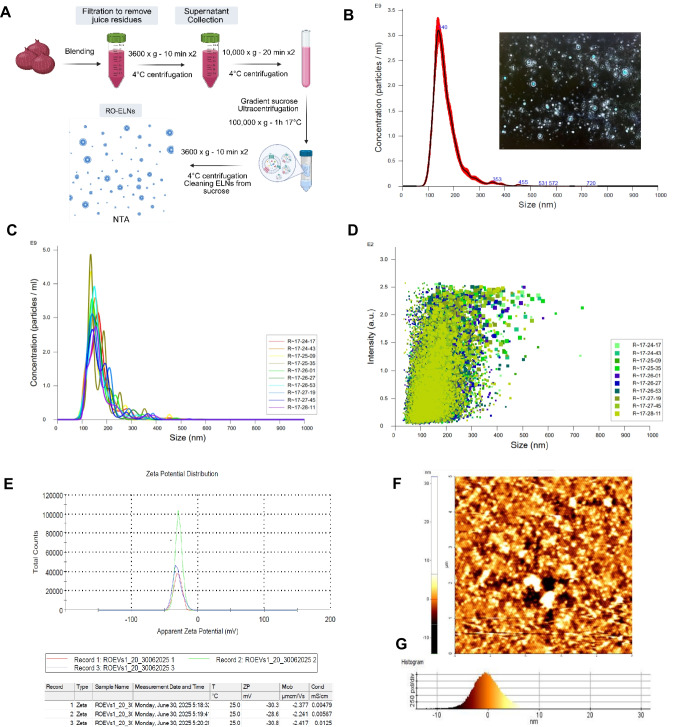
Purification and characterization of red onion–derived ELNs. **A** Illustrative scheme of the UC approach for isolating RO-ELNs. **B** RO-ELNs size and concentration were analyzed using NTA. **C**, **D** The hydrodynamic size distribution and intensity of RO-ELNs. **E** The surface charge of RO-ELNs using zeta sizer nano ZS. **F**, **G** Morphology of RO-ELNs and their intensity were analyzed using AFM

ELNs were purified from red onion juice using sucrose density gradient ultra-high-speed centrifugation (Fig. 1A). This technique enables the separation and collection of ELNs based on their density. The isolated ELNs were analyzed for their concentration, size distribution, and intensity using NTA, which visualizes and tracks the random movement of individual nanoparticles in a liquid using a laser microscope. The findings showed that RO-ELNs yielded 2.42 × 10^11^ particles/mL and are small-sized, about 169 nm in diameter, as shown in (Fig. 1B), which aligns with the size guidelines set by the International Society for Extracellular Vesicles [46]. The hydrodynamic size distribution and intensity data of RO-ELNs demonstrate that they are relatively uniform in size, indicating a high degree of homogeneity [10] as observed in (Fig. 1C, D). RO-ELNs have a ζ-potential of approximately −30 mV as indicated in (Fig. 1E), measured by DLS, showing their stable, negatively charged nature through light scattering. AFM analysis further validates the presence of isolated RO-ELNs by examining their morphology and biophysical characteristics, as shown in (Fig. 1F, G).

### Time Dependent Internalization of RO-ELNs by HMC3 Cells

**Fig. 2:**
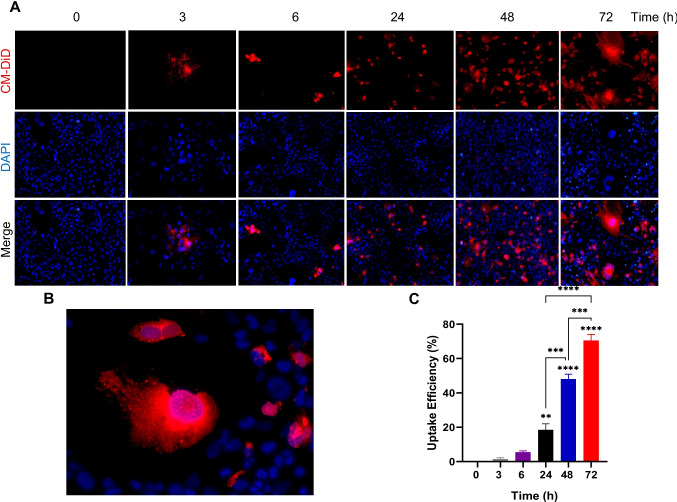
RO-ELNs were internalized by HMC3 cells. **A** The internalization of CM-DiD labeled RO-ELNs at several time points (0, 3, 6, 24, 48, 72) h was observed by fluorescent microscopy. **B** A higher magnification image illustrating the uptake of RO-ELNs by HMC3 cells at 48 h. **C** Measurement of uptake efficiency of RO-ELNs by HMC3 for 3, 6, 24, 48, 72 h. Data are expressed as mean ± standard (*n* = 2): **P < 0.01; ***P < 0.001; ****P < 0.0001

To assess the cellular uptake of CM-DiD-labeled RO-ELNs by human microglial HMC3 cells, the nanovesicles were incubated for 0, 3, 6, 24, 48, and 72 h and analyzed using fluorescence microscopy at 10× magnification, as shown in (Fig. 2A). The labeling confirmed the RO-ELNs’ lipid composition and successful internalization. Early detection within HMC3 cells occurred at 3 h post-incubation, with uptake steadily increasing over time (Fig. 2C). After 24 h, approximately 20% of cells displayed intracellular RO-ELNs signals, 50% at 48 h, rising to 70% by 72 h, indicating a sustained and efficient internalization process (Fig. 2C). Images of RO-ELNs internalization captured at 24, 48, 72 h at 5× magnification are illustrated in (Fig. S1). High-magnification imaging at 20× (Fig. 2B) indicates that RO-ELNs are internalized by the cells; however, due to the resolution limits of the current imaging method, it is unclear if the fluorescent signals represent actual intracellular localization or surface adherence. Thus, while our findings suggest cellular absorption, they demand additional exploration using higher-resolution imaging methods to validate precise intracellular localization.

### HPLC-Based Component Profiling of Red Onion Samples

**Fig. 3:**
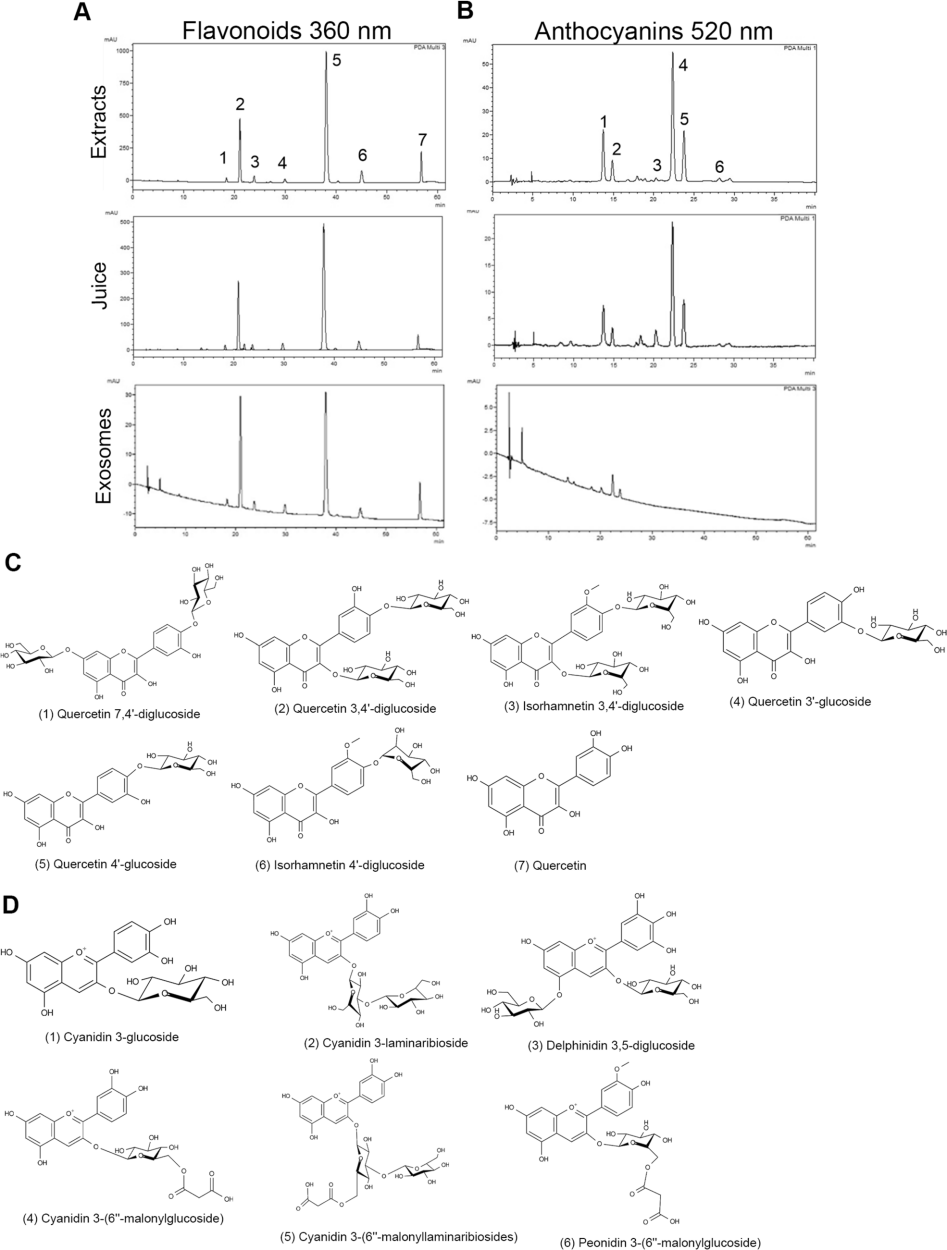
Chromatogram analysis of red onion samples: **A** Flavonoid (360 nm) components 1–7 were identified by their **C** structural profiles. **B** Anthocyanin (520 nm) components from 1 to 6 were identified by their **D** structural profiles

Early studies have demonstrated that red onions contain higher levels of antioxidant and anti-inflammatory flavonoids compared to yellow and white varieties [56]. The ethanolic extraction of red onion bulbs, prepared as detailed in the “Materials and Methods” section, produced a concentrated, syrupy purplish-red extract. HPLC analysis was performed to identify the phytochemicals in exosome-like nanovesicles, extract as well as red onion juice from which ELNs were isolated. The chromatogram showed that the extract contained relatively high levels of primarily quercetin derivatives, such as quercetin 3,4′-diglucoside and quercetin 4′-glucoside (Fig. 3A), and anthocyanins, particularly cyanidin 3-(6″-malonylglucoside) and other compounds like cyanidin 3-glucosides and cyanidin 3-(6″-malonyllaminaribiosides) (Fig. 3B). The elution pattern and retention sequence of major peaks matched those reported in previous studies [34, 40], confirming the presence of characteristic anthocyanins and flavonols.

### Impact of RO-ELNs and Extract on the Viability and Growth of HMC3 Cells

**Fig. 4:**
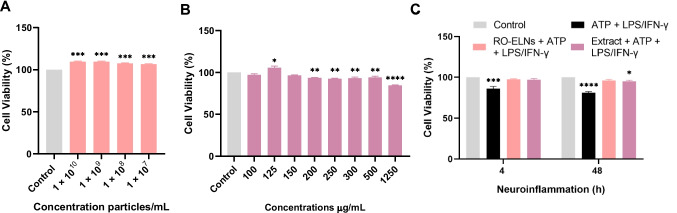
Cytotoxicity effects of RO-ELNs and extracts on HMC3 cells and inhibition of LPS-induced HMC3 death. **A**, **B** HMC3 were supplemented with RO-ELNs and extracts with different concentrations (1 × 10^10^–1 × 10^7^ particles/mL) extract (100–1250 µg/mL) for 24 h, and the cell viability was assessed. **C** The ability of RO-ELNs and extract to increase HMC3 cells’ viability when treated with ATP + LPS/IFN-γ at 4 and 48 h. Data are expressed as mean ± standard (*n* = 3): *P < 0.05; **P < 0.01; ***P < 0.001; ****P < 0.0001

To evaluate the cytotoxicity of ELNs and their corresponding extract, HMC3 cells were treated with varying concentrations of ELNs (1 × 10^10^ to 1 × 10^7^ particles/mL) and extract (100–1250 µg/mL), and then cell viability was analyzed after 24 h using the MTT assay. Results showed no cytotoxic effects of RO-ELNs at any concentration (Fig. 4A), with the cell viability increasing to 107–110% as their concentration increased. In contrast, the extract showed higher cell viability at 125 µg/mL, but the viability decreases with increasing concentration (Fig. 4B). A concentration of 1 × 10^9^ particles/mL of RO-ELNs, as well as 125 µg/mL of extract, was used for subsequent experiments. Then a combination of ATP + LPS/IFN-γ reduced cell viability by 85% at 4 h of neuroinflammation model. However, pretreatment with RO-ELNs and extract preserved cell viability, keeping it closer to control levels. These findings demonstrate that red onion extract does not negatively impact cell viability at optimal concentrations, supporting its safety and potential as a phytochemical source for therapeutic applications.

### TIME-DEPENDENT Effects of LPS/IFN-γ-Induced Neuroinflammation on ROS Production, NLRP3 Expression, and Cytokine Secretion in HMC3 Cells

**Fig. 5:**
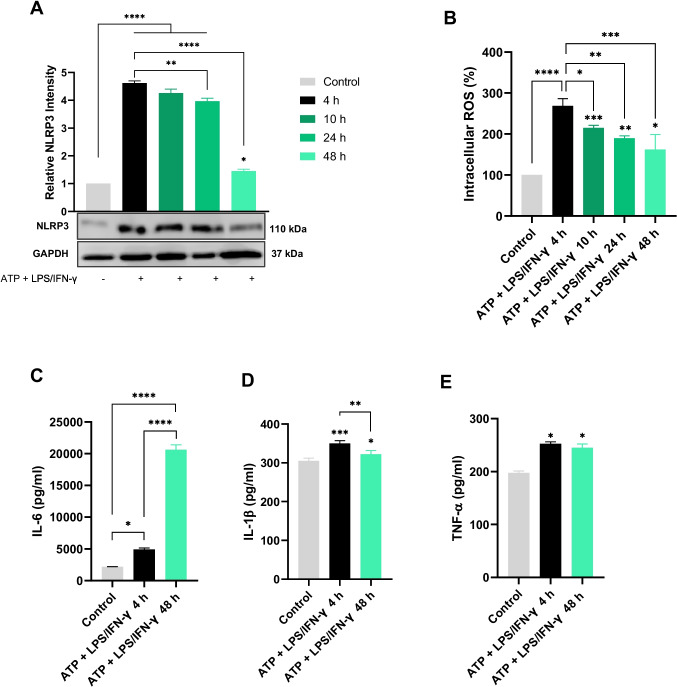
ATP + LPS/IFN-γ triggers a time-dependent inflammatory response marked by increased ROS, NLRP3 expression, and IL-6, TNF-α, and IL-1β secretions. **A**, **B** Relative NLRP3 intensity and ROS production at various time points 4, 10, 24, 48 h. **C**, **E** IL-6, TNF-α, and IL-1β secretions at 4 and 48 h as these hours of LPS treatment were selected for further analysis. The data shows the progression from acute to prolonged inflammation, with significant increases in NLRP3 expression, ROS, IL-1β production at early hours, and IL-6 secretion in late hours of inflammation. Data are expressed as mean ± standard (*n* ≥ 3): *P < 0.05; **P < 0.01; ***P < 0.001; ****P < 0.0001

To differentiate models of inflammation duration, the time-dependent expression of the NLRP3 inflammasome was evaluated. The NLRP3 inflammasome is a cytoplasmic multiprotein complex that serves as a crucial regulator of innate immune signaling in microglia as well as the synthesis of pro-inflammatory cytokines, including IL-1β (W. [52]). Microglia were preexposed to a danger signal ATP followed by LPS and IFN-γ stimulation at various time intervals. Western blot analysis of protein lysates from HMC3 cells assessed NLRP3 expression, capturing strong NLRP3 expression within the first 4 h of inflammation mimicking acute inflammation, followed by a steady drop beginning at 24 h, culminating in suppression at 48 h indicating a shift to prolonged inflammation (Fig. 5A). Correspondingly, NLRP3 acts as an upstream regulator of IL-1β; therefore, changes in NLRP3 expression are reflected in the secretion levels of IL-1β. Thus, IL-1β secretion peaked at 4 h and decreased by 48 h (Fig. 5E), confirming that the NLRP3 inflammasome complex shows strong activation in early acute neuroinflammation (around 4 h) but becomes suppressed in persistent inflammation (48 h). ROS levels were assessed using DCFH-DA. The findings demonstrated that HMC3 cells subjected to ATP + LPS/IFN-γ for 4 h exhibited a significantly elevated intracellular ROS concentration compared to subsequent time points, where a gradual decrease was observed at 10 and 24 h. After 48 h of treatment, oxidative stress diminished but remained consistently increased relative to baseline (Fig. 5B).

Conversely, cytokine detection assay results indicated that IL-6 exhibited minimal expression initially, but levels rapidly escalated by 48 h, demonstrating its role in the sustained inflammatory response (Fig. 5C). TNF-α demonstrated no significant variation between these two time periods, indicating a more stable yet less dynamic role in the inflammatory process, in accordance with preliminary findings from our laboratory [53].

### RO-ELNs Necessitate a Prolonged Incubation Duration, Unlike Extract Which Requires Only a Brief Period to Inhibit NLRP3 Inflammasome Activation

**Fig. 6:**
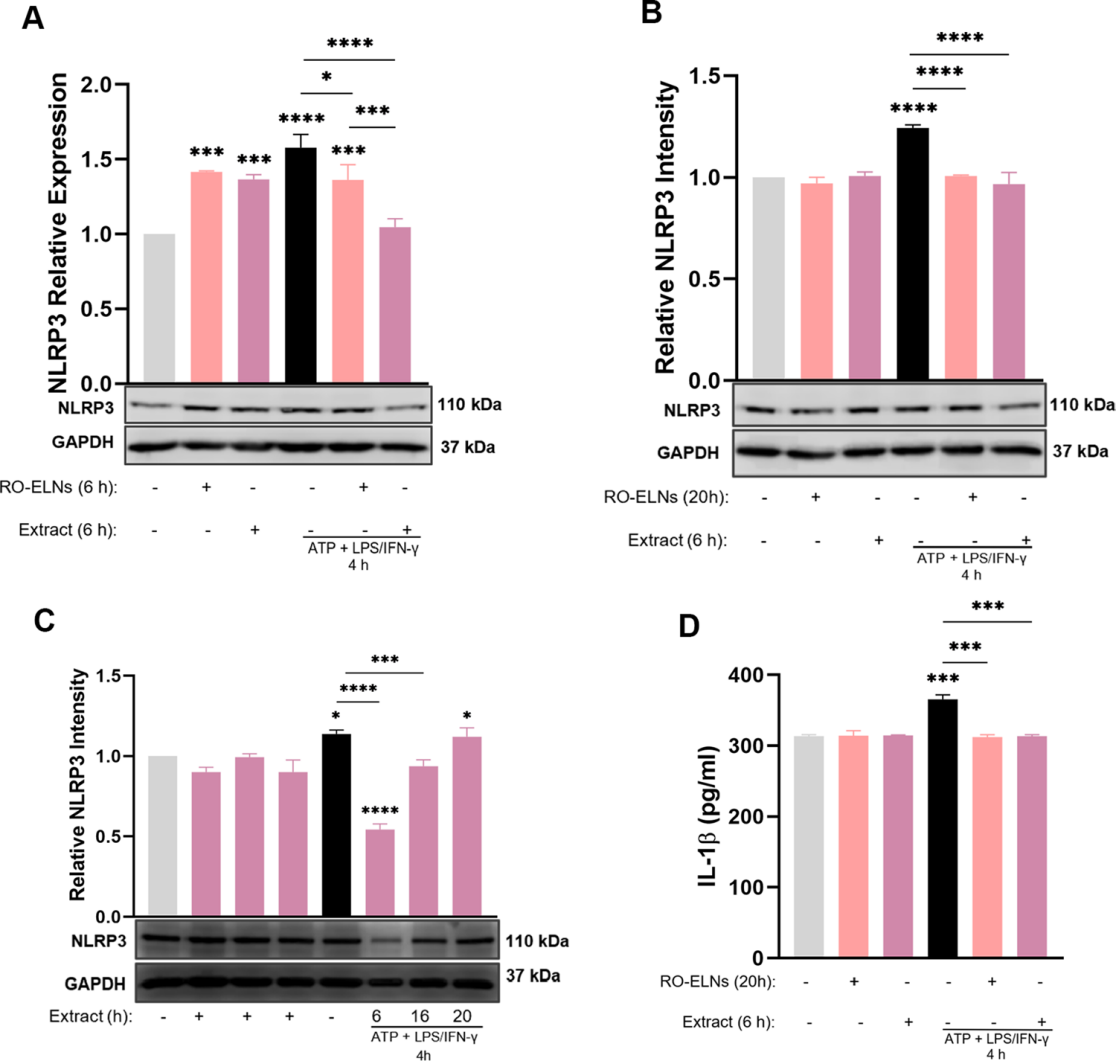
Assessment of different pre-incubation times of RO-ELNs vs. extract on inhibiting NLRP3 inflammasome activation. **A** HMC3 were pretreated with 1 × 10^9^/mL of RO-ELNs for 6 h and **B** for 20 h as well as 125 µg/mL of extract for 6 h followed by ATP+LPS/IFN-$\gamma$ to activate NLRP3 inflammasome. **C** HMC3 were pretreated with 125 µg/mL of extract for (6, 16, 20) h followed by ATP+LPS/IFN-$\gamma$. GAPDH was used as housekeeping protein. **D** The ability of RO-ELNs and extract to decrease IL-1β. Data are expressed as mean ± standard (*n* = 2): *P < 0.05; ***P < 0.001; ****P < 0.0001

To investigate the ability of RO-ELNs and the extract in inhibiting NLRP3 inflammasome activation in HMC3 cells in further analysis, initially either 1 × 10^9^ particles/mL of RO-ELNs or 125 µg/mL of extract was incubated with HMC3 cells for 6 followed by a 4 h stimulation induced by ATP + LPS/IFN-γ. The findings indicate that 6 h of extract pretreatment strongly suppressed NLRP3 expression, as indicated in (Fig. 6A-B-C). On the other hand, RO-ELNs slightly suppressed NLRP3 expression at 6 h pretreatment (Fig. 6A), but their notable inhibitory impact was observed after 20 h of pretreatment (Fig. 6B). This suggests that ELNs mainly necessitate prolonged incubation for efficient internalization to achieve anti-inflammatory actions, as enhanced uptake noted at 24 h with 20% efficiency which is adequate for therapeutic efficacy (Fig. 2A-C). The extract demonstrated that a shorter incubation period results in a swift reduction of NLRP3 expression due to accelerated intracellular metabolism and therapeutic efficacy, which subsequently diminishes with extended incubation time affecting their stability and effectiveness in cell culture (Fig. 6C). Therefore, western blot analyses revealed that RO-ELNs and extract could markedly inhibit the activation of HMC3 cells by reducing NLRP3 expression levels at acute stage (Fig. 6B) leading to reflected decrease in IL-1β secretion levels (Fig. 6D) indicating NLRP3 inflammasome suppression.

### Dual Protective Properties of RO-ELNs and Extract: Antioxidant and Anti-inflammatory Actions of RO-ELNs and Extract Against ROS and Pro-inflammatory Mediators and Cytokines in LPS/IFN-γ-Induced Neuroinflammation

**Fig. 7:**
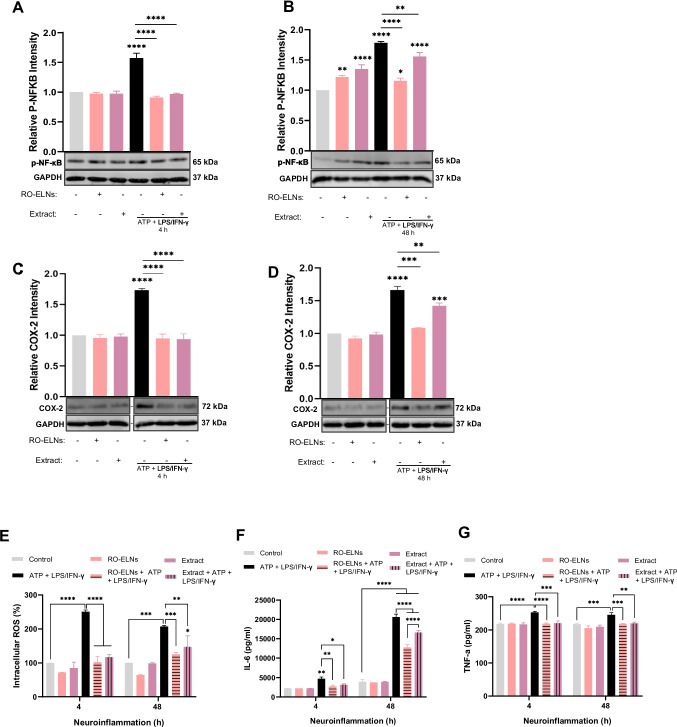
Anti-inflammatory and antioxidant properties of RO-ELNs and extract against ATP + LPS/IFN-γ stimulated pro-inflammatory mediators in HMC3 cells. RO-ELNs with a concentration of 1 × 10^9^ particles/mL were pretreated for 20 or 6 h before acute or prolonged neuroinflammation models. Extract with a concentration of 125 µg/mL was incubated for only 6 h for both acute and prolonged neuroinflammation models. Western blot and quantitative analyses show the ability of RO-ELNs and extract in inhibiting P-NF-κB (**A**, **B**) and COX-2 (**C**, **D**) expression levels in HMC3 cells at different time points 4 and 48 h of neuroinflammation. GAPDH was used as a housekeeping protein. The antioxidative properties of RO-ELNs and extract (**E**) and their ability to decrease IL-6, TNF-α secretion levels (**F**, **G**) at 4 and 48 h of neuroinflammation. Data are expressed as mean ± standard (*n* ≥ 3): *P < 0.05; **P < 0.01; ***P < 0.001; ****P < 0.0001. As a result of the initial reversed loading sequence for COX-2, lanes were rearranged digitally to align with the experimental grouping presented in the paper. All lanes originated from the same membrane and exposure. A separation dash marks the realignment. Uncropped original membranes can be found in Supplementary Figure

To further validate the previous observation, additional experiments were performed. The impact of RO-ELNs and extract at acute and prolonged stages was compared by testing different inflammatory mediators. For the acute phase module, HMC3 cells were pretreated with either (1 × 10^9^ particles/mL) RO-ELNs for 20 h or 125 µg/mL of extract hours and then stimulated with ATP + LPS/IFN-γ treatment for 4 h, as this was the optimized condition. For the persistent phase module, HMC3 cells were pretreated with RO-ELNs or extract for 6 h and then stimulated with ATP + LPS/IFN-γ treatment for 48 h. Activation of microglia triggers immune responses and leads to downstream signaling pathways that phosphorylate and activate targets such as NF-κB, enabling its translocation into the nucleus to upregulate various pro-inflammatory mediators such as NLRP3, COX-2, IL-6, IL-1β, TNF-α, and ROS [37]. Hence, RO-ELNs and extract were also assessed for their inhibitory effects on P-NF-κB. Western blot results revealed that both RO-EL and extract showed comparable inhibitory effects of 78–74% when combined with ATP + LPS/IFN-γ in the early stage of inflammation. Later phase of inflammation revealed that RO-ELNs had an inhibitory effect of 64% and extract 44% (Fig. 7A, B). During inflammation, the enzyme COX-2 plays a role by generating prostaglandins, which are substances that contribute to the sensations of pain and the development of swelling [50]. The expression of COX-2 was also evaluated in this study to ascertain the impact of RO-ELNs and extract on neuroinflammation (Fig. 7C, D. Interestingly, at the acute stage, both RO-ELNs and extract highly diminished COX-2 expression levels to 97–94% when combined with ATP + LPS/IFN-γ, but as inflammation progressed, RO-ELNs activity continued to exert regulatory effects and showed 58% decrease, while extract activity was influenced negatively and decreased the expression level only to 23%. DCFH-DA assay indicates that RO-ELNs and extract efficiently neutralized free radicals during acute inflammation; however, as inflammation progressed, extract began to lose its protective efficacy, while RO-ELNs sustained its inhibitory effect (Fig. 7E). LEGENDplex assay was used to detect cytokine levels to further validate the actions of RO-ELNs and extract. Under acute inflammatory condition, RO-ELNs and extract exhibited a comparable attenuating effect on IL-6 secretion when combined with ATP + LPS/IFN-γ. Although RO-ELNs showed better inhibitory effect when compared to the extract in prolonged inflammation, both RO-ELNs and extract did not significantly reduce IL-6, which increased rapidly in later hours, which could be a strong indication that they both follow the same mechanism of action (Fig. 7F). In addition, RO-ELNs and extract have similar inhibitory behavior on TNF-α secretion levels at both acute and persistent inflammation points (Fig. 7G).

## Discussion

In this research, organically cultivated red onions were preferably chosen for ELN extraction because they produce a higher concentration of RO-ELNs and demonstrate stronger antioxidant properties compared to their conventionally grown counterparts [31]. Ultracentrifuged red onion juice yielded a high concentration of RO-ELNs, which are nanosized particles and exhibit a spherical shape and a high negative surface charge (Fig. 1A-G). This negative charge maintains RO-ELNs dispersion stability through repulsive forces and influences their physicochemical activities [7, 36]. Red onion components like extract, juice, and ELNs demonstrated the presence of polyphenols, primarily quercetin and anthocyanin constituents as detected by HPLC (Fig. 3), which have been proven to possess anti-neuroinflammatory capacities against brain diseases [20, 29]. Although RO-ELNs contained lower flavonoid levels than extract and juice, their antioxidant and anti-inflammatory activities are primarily attributed to the presence of bioactive compounds like lipids, as indicated (Fig. 2).

Given that RO-ELNs exhibited a surface charge of about −30 mV, this is likely to influence their interaction with cellular membranes and consequent biological responses. Their notable uptake by HMC3 cells (Fig. 2 and Fig. S1) reinforces the earlier report that negatively charged nanoparticles are typically more readily taken up by phagocytic cells like macrophages [16]. Factors such as lipid composition, a size < 200 nm, and a ζ-potential of −30 mV may enhance RO-ELNs uptake by facilitating vesicle transport. However, a limitation of this study is that the suggested mechanisms regarding the sustained release, bioavailability, and lipid-mediated actions of RO-ELNs were not directly tested. Consequently, these interpretations are speculative and should be validated through dedicated pharmacokinetic and intracellular trafficking studies in future research. The intracellular localization is most probably guided by surface proteins, directing RO-ELNs to cellular trafficking allowing RO-ELNs to exert their pharmacological effects [30]. However, due to the lack of confocal microscopy or other high-resolution techniques, the precise subcellular location remains speculative. As a result, our findings mostly indicate the presence of cellular absorption rather than precise localization inside specific cellular compartments.

The HPLC analysis revealed that juice had relatively lower flavonoid contents compared to the extract, while RO-ELNs exhibited the lowest levels (Fig. 3A, B), consistent with a report that nanovesicles derived from *Lycium ruthenicum* contain fewer anthocyanins than their source tissues [57]. Extra washing steps of ELNs after ultracentrifugation are recommended before HPLC analysis to further confirm their metabolite contents.

Given the non-cytotoxic nature of herbal exosome-like nanovesicles [11], a possible explanation is that the negatively charged nanovesicles have weaker interactions with cells, resulting in lower cytotoxicity, in contrast to positively charged vesicles that tend to be more cytotoxic [43]. Thus, the ζ-potential of RO-ELNs likely influenced their low cytotoxicity (Fig. 4A). The lack of cytotoxicity in ELNs derived from red onions is consistent with previous findings on lower cytotoxicity of extracellular vesicles derived from regular onions [25]. Extract, on the other hand, showed a decrease in cell viability with higher doses (Fig. 4B), indicating that polyphenols and flavonoids in the extract can act as antioxidants at lower doses and pro-oxidants at higher doses, triggering ROS production and cell death [42]. Previous studies have shown that *Allium cepa *L. extract demonstrated the lowest cytotoxicity and high safety profiles at therapeutic concentrations and strong pathogen selectivity compared to other tested plant extracts [1]. Consistent with this, our findings confirm that the red onion extract does not adversely affect cell viability at the selected optimal concentration, supporting its potential as a safe phytochemical source for therapeutic applications.

Two models for the time-course assessment of neuroinflammation were designed based on NLRP3 inflammasome activity. Neuroinflammation initiates when LPS binds to TLR4 on microglia, activating the innate immune response and inducing inflammatory signals that stimulate the nuclear translocation of the key transcription factor NF-κB, resulting in the activation and transcription of inflammatory mediators such as NLRP3, COX-2, ROS, IL-1β, IL-6, and TNF-α. NF-κB activation is a key feature in patients with NDDs and has been associated with cognitive decline. The signaling of NF-κB is crucial for the activation of the downstream NLRP3 inflammasome, which is intricately associated with the innate immune response as strong expression of NLRP3 was captured at 4 h (Fig. 5A) and inflammatory responses. NLRP3 inflammasome activation is often triggered by Aβ or α-syn, contributing to the pathology of AD and PD [2]. In this work, ATP preconditioning acts as an early activation signal, imitating extracellular ATP produced by dying cells in NDD patients. Pre-exposure primes microglia, amplifying their pro-inflammatory response to LPS-IFN-γ stimulation, activating NF-κB, and potentiating inflammasome assembly. The activation of the NLRP3 inflammasome, which is frequently triggered by pathogenic factors such as Aβ or α-synuclein in Parkinson’s and Alzheimer’s diseases, results in pyroptotic neuronal death, toxic protein accumulation, severe neuroinflammation, and the cleavage and release of IL-1β [52]. Therefore, targeting NLRP3 may offer a promising strategy to mitigate microglia-driven neuroinflammation and enhance clearance of pathogenic proteins. The sequential application of ATP followed by LPS/IFN-γ in this study strongly activated the NLRP3 inflammasome, aligning with the findings of [33] which demonstrated that ATP preconditioning primes microglia-like cells via epigenomic and transcriptome reprogramming. This priming amplifies the pro-inflammatory response to future LPS stimulation, indicating immunological conditioning related to microglial states associated with epilepsy and potentially contributing to neuroinflammation in NDDs.

Immune cells employ negative feedback mechanisms to suppress NLRP3 inflammasome activation during prolonged inflammation such as at 48 h to prevent excessive cellular damage (Fig. 5A). In addition, ROS levels at 4 h were elevated compared to those at 48 h of inflammation, where this gradual decline reflected the immune system adaptability to avert further oxidative damage (Fig. 5B) [9]. Accordingly, two-time intervals, 4 and 48 h, were utilized to model acute and prolonged neuroinflammation for further analyses (Fig. 5).

RO-ELNs and extract were assessed for their capacity to suppress NLRP3 expression after pretreatment. The slight reduction in NLRP3 expression at 6 h of RO-ELNs pretreatment suggests that nanovesicles may exert early protective, extracellular anti-inflammatory effects prior to substantial cellular internalization (Fig. 6A), whereas 20 h revealed enhanced suppression of the NLRP3 inflammasome due to increased internalization of ELNs (Fig. 6B, D Conversely, pretreatment with extract for 20 h showed an effect opposite to that of RO-ELNs (Fig. 6C). This suggests that the stability of red onion extract polyphenols may experience structural alterations and degradation under cell culture conditions within 24 h, potentially affecting their bioavailability and experimental results [3, 32]. Future assessments of cleaved caspase-1 and ASC speck formation may enhance the comprehension of the mechanistic insights into the influence of RO-ELNs and the extract on neuroinflammatory responses.

The persistence of P-NF-κB and IL-6 in prolonged inflammation (Fig. 7A, B, F) further underscores the need for therapeutic nanovesicles, which can more effectively modulate this pathway compared to the extract. The presence of lipids and proteins in RO-ELNs could contribute to their anti-inflammatory characteristics [48, 49]). In fact, the presence of aromatic hydroxyl groups found in quercetins and anthocyanins (Fig. 3C, D), as well as the positive charges found on the oxygen of the pyran ring of anthocyanins, render red onion extract with strong anti-oxidant properties [13, 17]. It has been confirmed that early incubation hours contributed to the extract’s inhibitory effect; however, their longer incubation time did not improve their biological activity like with RO-ELNs. Therefore, their stability is negatively affected by longer incubation duration and other conditions. The flavonoids in the extract can be hydrolyzed and undergo structural transformations due to digestive enzymes produced by cells or present in the media, as well as alterations in pH, heat, and oxidative reactions during incubation time [32], [55]. Therefore, the fate of quercetins, anthocyanins, and their metabolites in the extract can be influenced by all these factors in this research.

## Conclusion and Future Perspectives

Overall, this study highlights that Liliaceae family member *Allium cepa* L., species, particularly red onion–derived materials such as extracts and exosome-like nanovesicles, retain the inherent biological efficacy of the original source. However, their biological efficiency differs according to formulation and treatment settings, as each form necessitates optimized use to attain optimal therapeutic results. RO-ELNs and extract as natural agents demonstrated significantly comparable antioxidant and anti-inflammatory properties in human microglial HMC3 cells during the acute neuroinflammation phase, with RO-ELNs revealing superior efficacy, especially in the prolonged inflammation phase due to their gradual cellular uptake and sustained controlled release characteristics of therapeutic molecules, which are facilitated by their lipid bilayer, further underscoring their therapeutic potential. Conversely, extract has experienced expedited intracellular metabolism, and its protective properties were adequate for shorter treatment time. These findings collectively suggest that red onion–derived exosome-like nanovesicles can serve as promising novel biomaterials for modulating neuroinflammation, a critical factor in NDDs. Further in vivo studies are recommended to confirm the therapeutic effects of RO-ELNs and the extract in neuroinflammation. Future research should explore their mechanisms of action, optimize their large-scale production from organic red onions, and evaluate different delivery methods for clinical use. Additionally, comparing RO-ELNs with other plant-derived ELNs could help identify their unique advantages as natural anti-inflammatory and antioxidant agents.

## Supplementary Information

Below is the link to the electronic supplementary material.

ESM 2 (PNG 2.79 MB)

High Resolution Image (TIF 3.70 MB)

ESM1 (PDF 227 KB)

## Data Availability

Data will be made available on a reasonable request.
