# Supplementary figures and images for: Comparative Analysis of Red Onion-Derived Exosome-Like Nanovesicles and Extract Reveals Sustained Immunomodulatory Effects in LPS/IFN-γ-Stimulated Microglia
Source: Mol Neurobiol. 2026 Mar 30;63(1):533. doi: 10.1007/s12035-026-05820-0 (PMC13035600; doi:10.1007/s12035-026-05820-0)

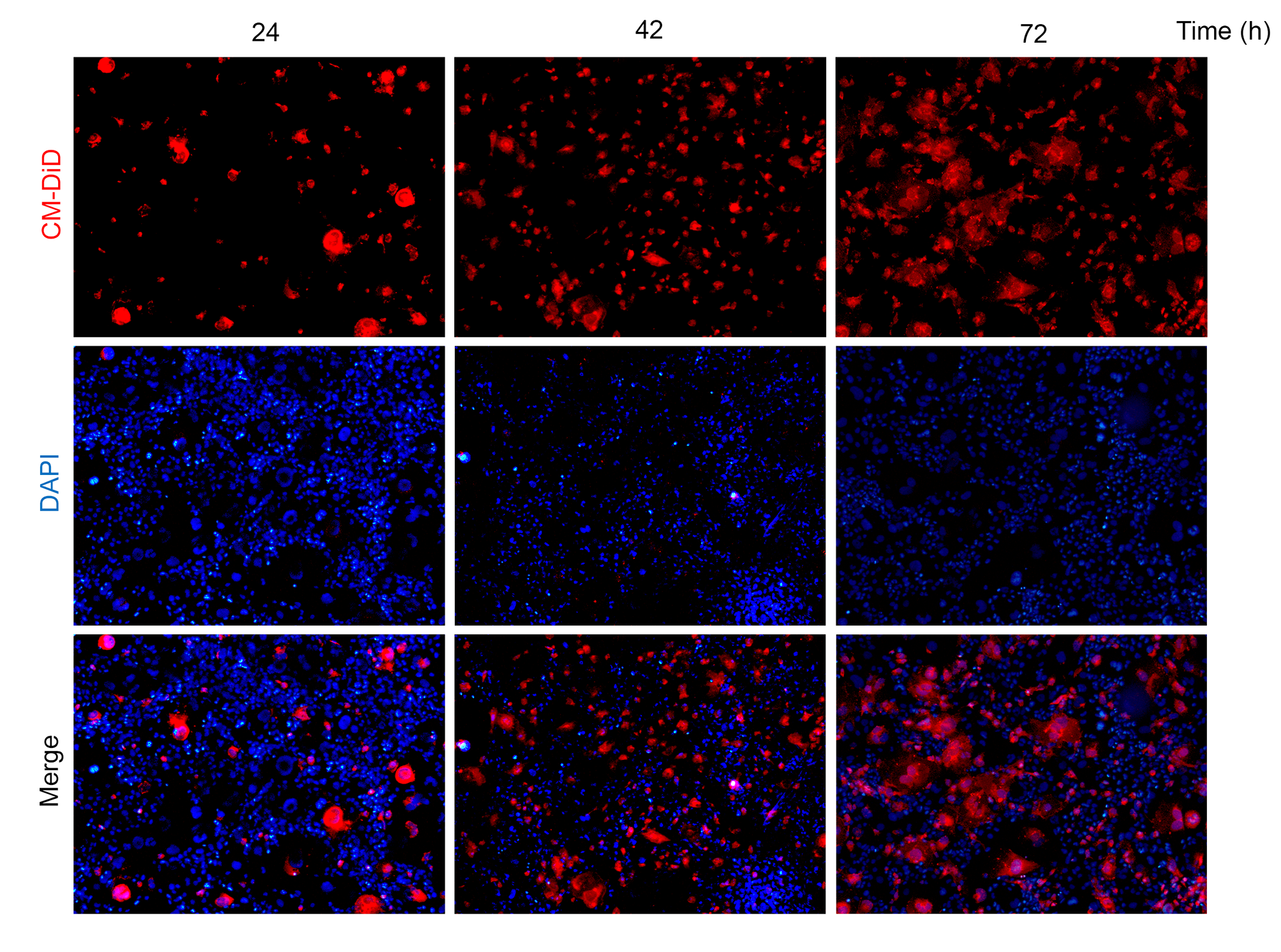

Supplement: Supplementary file 1 — (PNG 2.79 MB) [file 12035_2026_5820_Fig8_ESM.png]

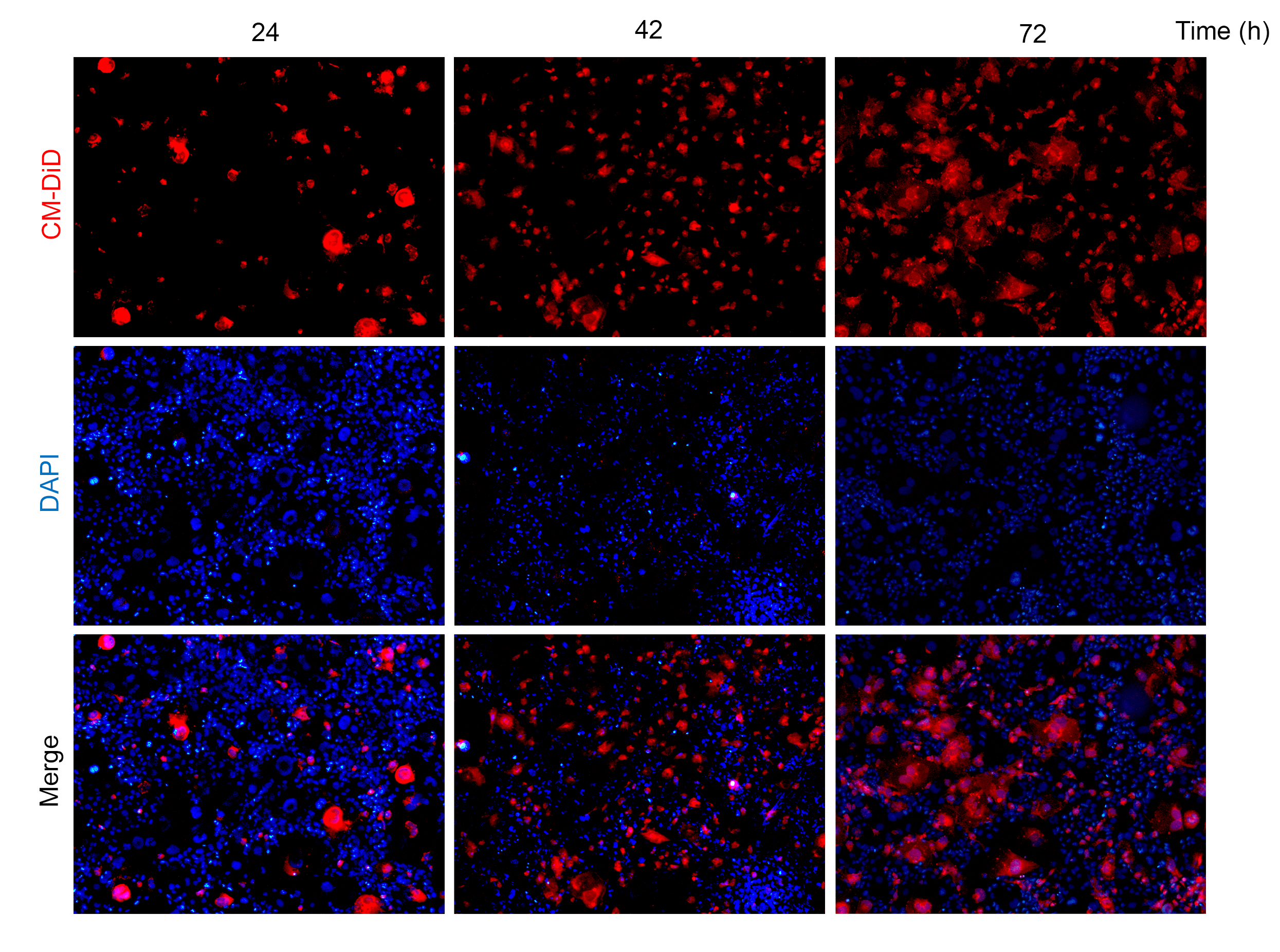

Supplement: Supplementary file 2 — High Resolution Image (TIF 3.70 MB) [file 12035_2026_5820_MOESM1_ESM.tif]

**Fig. 5**

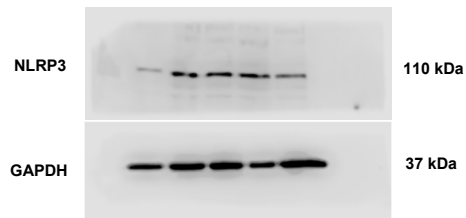

**Fig. 6**

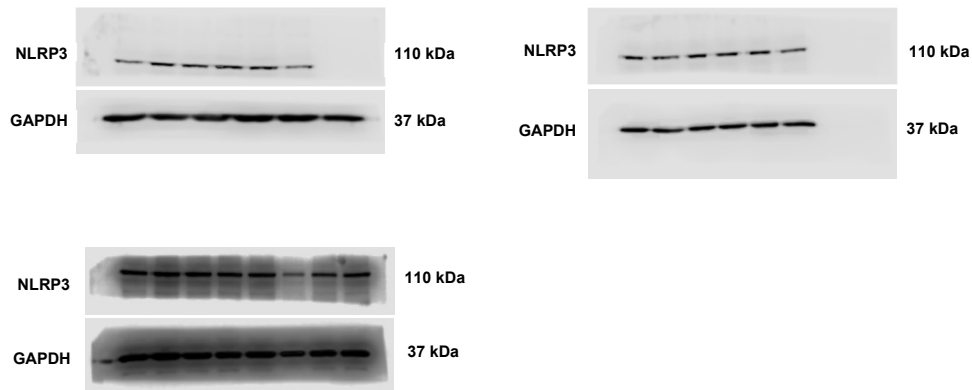

**Fig. 7**

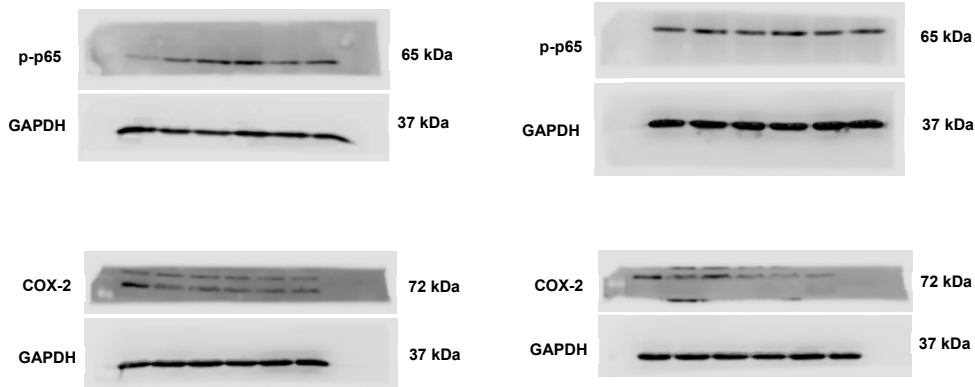

Supplement: Supplementary file 3 — (PDF 227 KB) [file 12035_2026_5820_MOESM2_ESM.pdf]
